# Augmenting Denver criteria yields increased BCVI detection, with screening showing markedly increased risk for subsequent ischemic stroke

**DOI:** 10.1007/s10140-019-01677-0

**Published:** 2019-02-12

**Authors:** Frank V. Bensch, Elina A. Varjonen, Tuomo T. Pyhältö, Seppo K. Koskinen

**Affiliations:** 1 Department of Radiology, Töölö Trauma Center, Helsinki University Hospital, HUS, Topeliuksenkatu 5, P.O. Box 266, 00029 Helsinki, Finland; 2 Department of Trauma Surgery, Töölö Trauma Center, Helsinki University Hospital, Helsinki, Finland; 3 Department of Radiology, Karolinska Institute, Stockholm, Sweden

**Keywords:** BCVI, Cerebrovascular injury, CT angiography, Screening, Trauma, Ischemic stroke

## Abstract

**Purpose:**

BCVI may lead to ischemic stroke, disability, and death, while being often initially clinically silent. Screening criteria for BCVI based on clinical findings and trauma mechanism have improved detection, with Denver criteria being most common. Up to 30% of patients do not meet BCVI screening criteria. The aim of this study was to analyze the effect of augmented Denver criteria on detection, and to determine the relative risk for ischemic stroke.

**Methods:**

Denver screening criteria were augmented by any high-energy trauma of the cervical spine, thorax, abdomen, or pelvis. All acute blunt trauma WBCT including CT angiography (CTA) over a period of 38 months were reviewed retrospectively by two Fellowship-trained radiologists, as well as any cerebral imaging after the initial trauma.

**Results:**

1544 WBCT studies included 374 CTA (m/f = 271/103; mean age 41.5 years). Most common mechanisms of injury were MVA (51.5%) and fall from a height (22.3%). We found 72 BCVI in 56 patients (15.0%), with 13 (23.2%) multiple lesions. The ICA was affected in 49 (68.1%) and the vertebral artery in 23 (31.9%) of cases. The most common injury level was C2, with Biffl grades I and II most common in ICA, and II and IV in VA. Interobserver agreement was substantial (Kappa = 0.674). Of 215 patients imaged, 16.1% with BCVI and 1.9% of the remaining cases had cerebral ischemic stroke (*p* < .0001; OR = 9.77; 95% CI 3.3–28.7). Eleven percent of patients with BCVI would not have met standard screening criteria.

**Conclusions:**

The increase in detection rate for BCVI justifies more liberal screening protocols.

## Introduction

Blunt cerebrovascular injuries (BCVI) are uncommon but potentially devastating injuries in blunt trauma patients. The most common trauma mechanisms are stretching, direct impact, or shearing forces in areas where vessels are either fixed in place or run close to rigid or bony neighboring tissues, thus mainly affecting the internal carotid (ICA) and vertebral arteries (VA) [1, 2].

Most BCVI are initially asymptomatic and hence easily missed in clinical examination [2–4]. Associated traumatic brain injury (TBI) is common and may mask the signs of a vascular injury. The majority of BCVI-related ischemic cerebral infarcts occur during the first couple of days after the injury, but may develop even after weeks or months [5, 6].

Morbidity in blunt carotid injury ranges between 32 and 67% and in blunt vertebral artery injury between 14 and 24%, whereas mortality ranges between 13 and 38% and 8–18%, respectively [1, 7]. The risk of stroke depends on the grade of the injury [8] and is higher in ICA than in VA injuries [2, 9, 10]. Early treatment is shown to be safe and reduce BCVI-related neurologic sequelae. Current treatment methods include antiplatelet and anticoagulation therapy as well as endovascular treatment in patients with contraindications for thrombolysis [5, 6, 11].

Before the implementation of systematic screening protocols, overall incidence for BCVI in blunt polytrauma was reported to be at 0.1–1% [2, 12, 13], and is now believed to be at 1–2% [14, 15]. With more efficient and liberal screening, markedly higher detection rates of up to 2.7–9% in blunt polytrauma patients have been reported [3, 13, 14, 16, 17].

Digital subtraction angiography (DSA) is the reference standard for imaging due to its high resolution and the option to evaluate collaterals [2, 14]. It has, in clinical practice, been replaced by CT angiography (CTA) with at least 16-slice scanners, which is non-invasive, widely available, and cost-effective [2, 14, 17–19].

A wide spectrum of screening criteria for BCVI has been suggested, the most common being the modified Denver criteria consisting of clinical signs, symptoms, and trauma mechanisms that have been shown to correlate with BCVI (Table 1) [13, 20, 21]. Nevertheless, to as much as 30% of the BCVI patients, any of these clinical or radiological risk factors indicating BCVI do not apply, and it has been suggested that more liberal screening protocols are required to detect BCVI in these patients [22–24]. CTA screening of high-energy trauma patients can easily be included into whole-body CT (WBCT) for trauma [4, 13, 16, 25].

**Table 1:** Denver screening criteria for BCVI

Denver screening criteria	
Signs/symptoms of BCVI	
Arterial hemorrhage	
Cervical bruit	
Expanding cervical hematoma	
Focal neurologic deficit	
Neurologic examination incongruous with head CT scan findings	
Stroke on secondary CT scan	
Risk factors for BCVI	
High-energy transfer mechanism with:	
LeFort II or III fracture	
Cervical-spine fracture patterns: subluxation, fractures extending into the transverse foramen, and fractures of C1–C3	
Basilar skull fracture with carotid canal involvement	
Petrous bone fracture	
Diffuse axonal injury	
Near hanging with anoxic brain injury	

The purpose of this study was to assess the detection rate for BCVI based on augmented Denver criteria in high-energy blunt trauma patients presenting in a single level-one trauma center, and to evaluate the relative risk for ischemic stroke for these patients.

## Materials and methods

This study was conducted in a level-one trauma center with a catchment area of 1.6 million people. The study population consists of patients admitted to the emergency department after high-energy deceleration trauma such as MVA or fall from a height greater than 4 m. During this study, there were two routine trauma-WBCT protocols in use, both including an initial non-enhanced cranial CT scan. The standard WBCT protocol (sWBCT) continues with a non-enhanced CT of the cervical spine, followed by the thorax in the arterial and subsequently the abdomen in the portal venous phase. The alternative angio-protocol consists of a dual-phased WBCT (dWBCT) including a first pass from orbits to ischium in the arterial phase, and a second pass of the upper abdomen in the portal venous phase (Table 2). The latter protocol thus includes an angiogram of the carotid and vertebral arteries for the purpose of BCVI screening, while exposing the patient to an increased radiation dose. The attending trauma surgeon decided on using dWBCT protocol based on modified Denver criteria, augmented by the addition of suspected cervical spine injury on any level, and any high-energy deceleration trauma with impact on chest, abdomen, or pelvis.

**Table 2:** Detailed description of the imaging protocols

	Standard WBCT (sWBCT)			Dual-phased WBCT (dWBCT)		Additional imaging (for both protocols)
Body part	Cervical spine	Thorax	Abdomen	Whole body	Upper abdomen	Urinal tract
Scan area	Skull base to jugulum	Jugulum to below diaphragm	Above diaphragm to ischium	Orbitae to ischium	Diaphragm to iliac crista	Diaphragm to ischium
Contrast enhancement	No contrast	Arterial phase	Venous phase	Arterial phase	Venous phase	After phase
Contrast delay (s)	–	25	65	25	65	300
Slice thickness (mm)	0.625	0.625	0.625	0.625	0.625	0.625
Reformatted series	ax/sag	ax/sag/cor	ax/sag/cor	CS: ax/sag/cor body: ax/sag/cor	ax/cor	ax/cor
Slice thickness (mm)	1.25/2.0	2.5/2.0/4.0		CS: 1.25/2.0 body: 2.5/2.5/4.0		2.0/2.0
Angio MIP	–	n	n	y	n	n
3D bone and vascular reformatted images	n	y	y	y	n	n

All WBCT were performed on a 64-row MDCT scanner (Discovery CT750 HD, GE Medical Systems, Milwaukee, WI, USA). All WBCT for blunt trauma between October 2011 and December 2014 were reassessed retrospectively using Agfa Impax picture archiving and communications system (PACS; Impax 6.5, Agfa Gaevert, Mortsel, Belgium). All sWBCT were excluded. Two board-certified and Fellowship-trained trauma radiologists (with 12 and 8 years of experience, respectively) independently reviewed all dWBCT studies with focus on the carotid and vertebral arteries blinded to initial results. All BCVI were graded using the Biffl classification [8] (Table 3). Artery, injury level, as well as concomitant injuries were documented and are summarized in Table 4. For interobserver agreement we used kappa statistics, with kappa-values 0.01–0.20 indicating slight agreement, 0.21–0.40 fair agreement, 0.41–0.60 moderate agreement, 0.61–0.80 substantial agreement, and 0.80–0.99 almost perfect agreement [26]. Additionally, if patients were subject to any cranial CT and/or MRI studies after the initial trauma, these images were retrieved and evaluated for ischemic lesions. Analysis was performed using IBM SPSS Statistics v.23 (IBM Corp., Armonk, NY, USA). IRB approval was obtained.

**Table 3:** Biffl classification for severity of BCVI

	Biffl classification
Grade I	Mild intimal injury or irregular intima with luminal narrowing < 25%
Grade II	Dissection with raised intimal flap, intraluminal thrombosis, or intramural hematoma with luminal narrowing > 25%
Grade III	Pseudoaneurysm
Grade IV	Luminal occlusion
Grade V	Transmural defect and active hemorrhage

**Table 4:** Associated injuries by body region in all patients imaged with dWBCT and in BCVI patients

Injury	*N*	% (/374)	*N*	% (of 56 BCVI patients)
Head	124	33.2	26	46.4
Cervical spine	45	12.0	15	26.8
Thorax	203	54.3	37	66.1
Abdomen	72	19.3	17	30.4
Pelvis	75	20.1	14	25.0

## Results

During 38 months, a total of 1544 WBCT studies were performed after blunt trauma, of which 374 included imaging of the craniocervical vessels in the arterial phase (m/f = 271/103; mean age 41.5 years). Most common mechanisms of injury were motor vehicle accident (MVA) (*n* = 192; 51.5%), fall from a height (*n* = 83; 22.3%), pedestrian vs. vehicle (*n* = 38; 10.2%), and bicycle accidents (*n* = 25; 6.7%). A total of 72 BCVI were identified in 56 patients (56/374; 15.0%), for an overall detection rate of 3.6% based on all trauma WBCT studies. Thirteen patients (13/56; 23.2%) had multiple BCVI. 68.1% of injuries (49/72) affected the internal carotid artery (ICA) and 31.9% (23/72) the vertebral artery (VA).

**Fig. 1:**
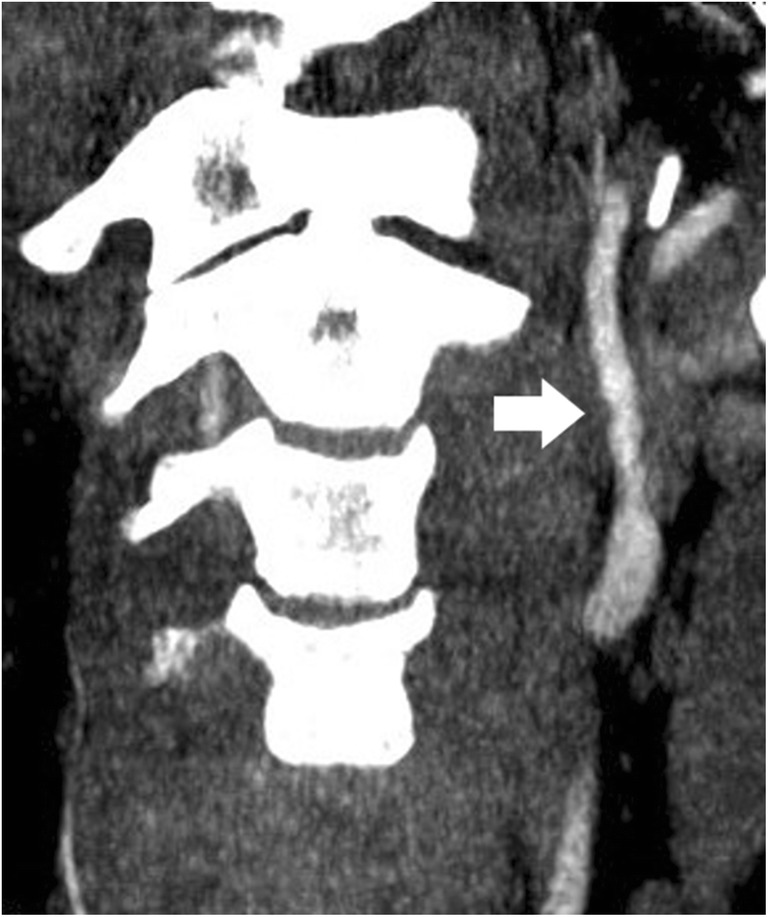
Grade I BCVI of the left ICA (arrow) in a 25-year-old female after MVA on CTA

**Fig. 2:**
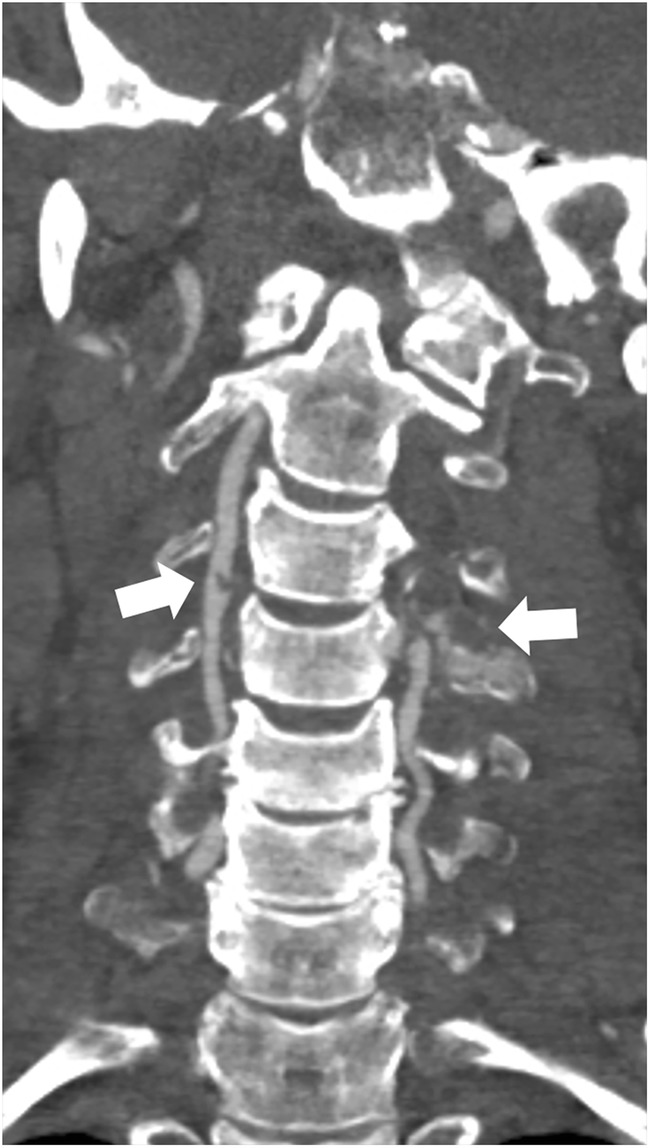
Grade II BCVI of the right vertebral artery and grade 5 BCVI of the left vertebral artery (arrows) on CTA in a 58-year-old male pedestrian who was hit by a car at 40 km/h

**Fig. 3:**
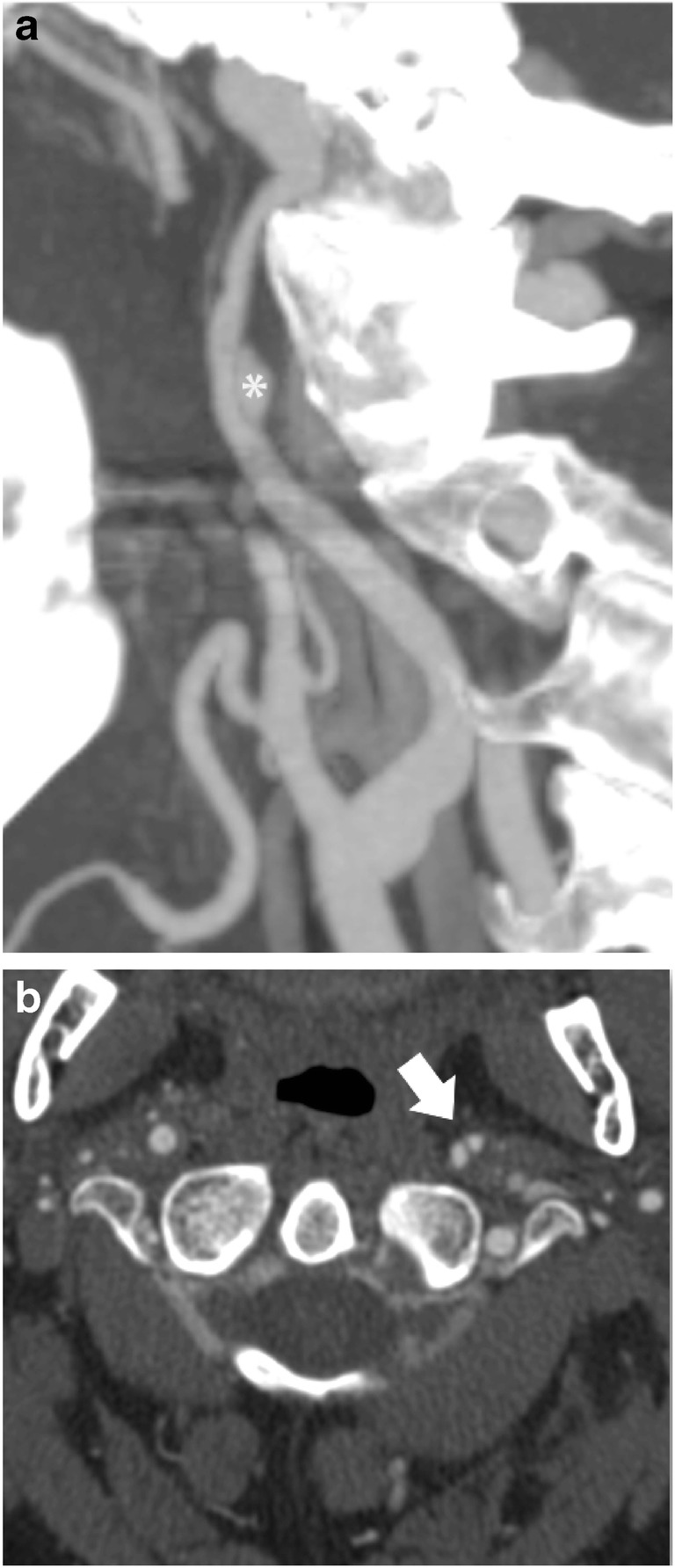
Grade III BCVI of the right ICA in a 51-year-old male after MVA. **a** Sagittal CTA MIP shows the outpouching of the lumen and arterial wall (asterisk) and **b** axial **CTA** images the double lumen indicating a pseudoaneurysm (arrow)

**Fig. 4:**
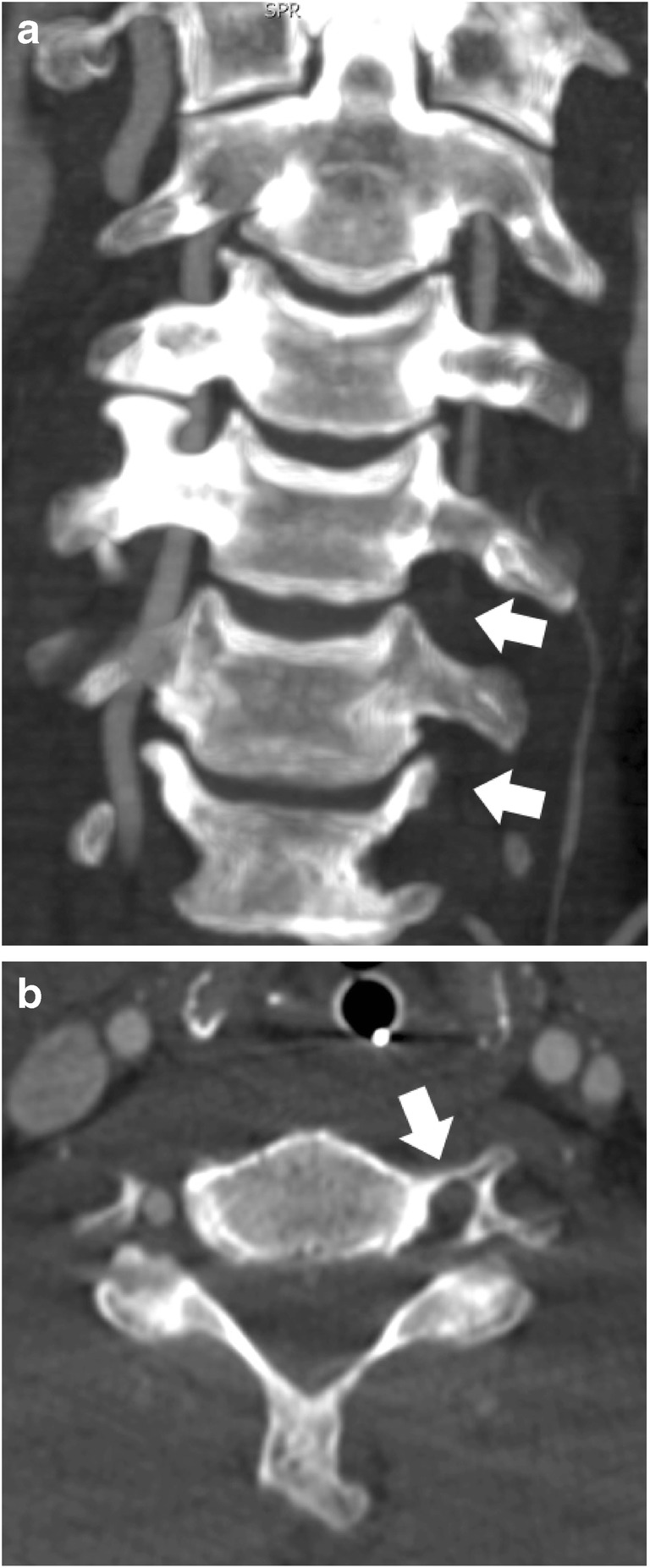
Grade IV BCVI of left VA on CTA in a 50-year-old male whose head hit the windshield in a MVA. **a** Coronal and **b** axial reformats show the absence of contrast in the lumen (arrows)

**Fig. 5:**
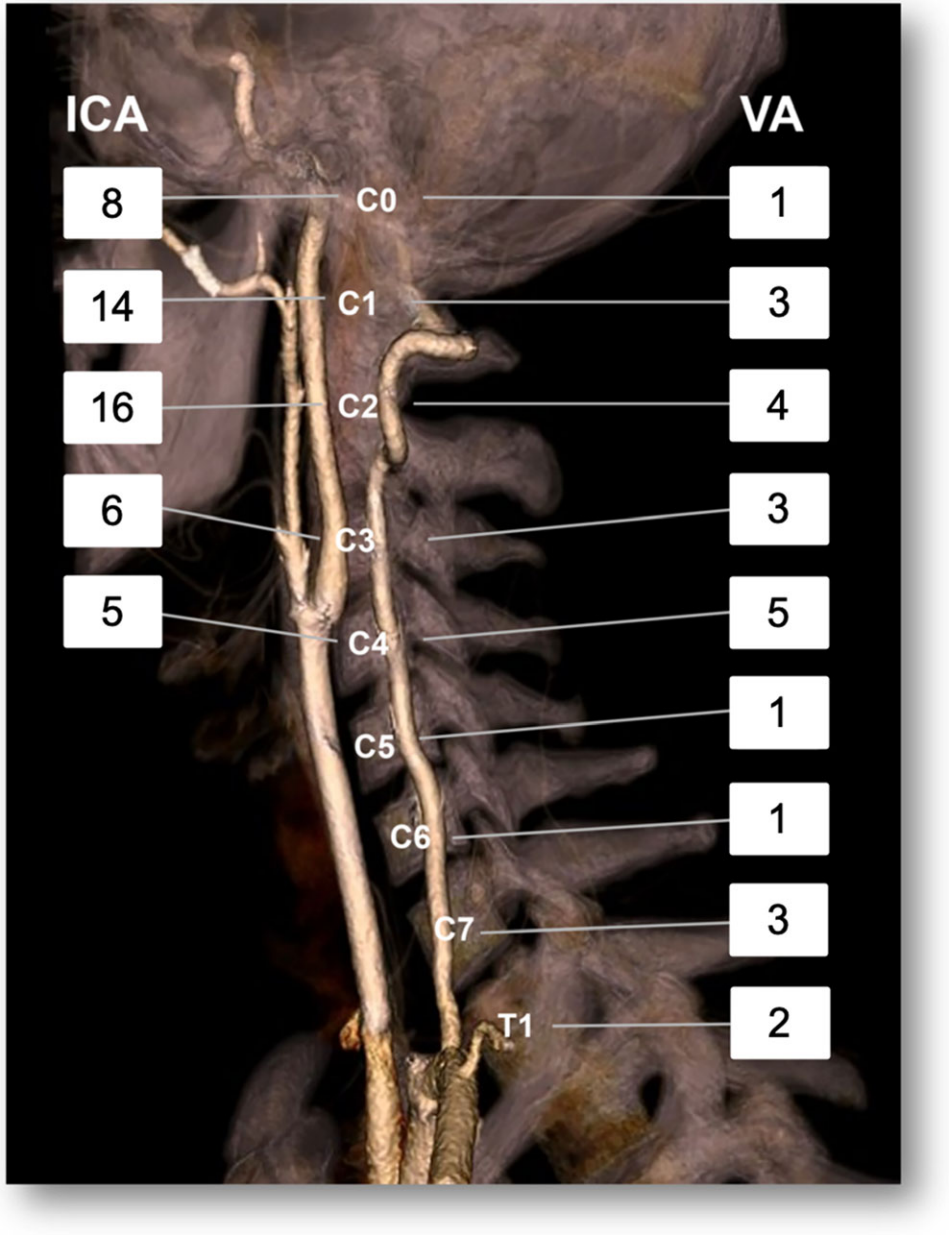
Number of BCVI by vertebral level in both ICA and VA on a surface-rendered CTA image without acute pathology

In ICA injuries, grades I and II and in VA injuries, grades II and IV were most common (ICA: G I 20/49; 40.8%; G II 15/49; 30.6%; G III 6/49; 12.2%; G IV 8/49; 16.3%; VA: G I 5/23; 21.7%; GII 8/23; 34.8%; GIII 2/23; 8.7% G IV 7/23; 30.4%; Figs. 1, 2, 3, and 4; Table 5). The most common level for BCVI was C2 in the ICA (16/49; 32.7%) and C4 in the VA (5/23; 21.7%). Figure 5 shows the number of BCVI by cervical vertebral level in both ICA and VA. Only a single grade V BCVI emerged, which was located in the basilar artery at the level of C0 (Table 6). In 3/56 (5.4%) of BCVI, there was clearly atherosclerosis present.

**Table 5:** The number of BCVI by Biffl grades in internal carotid arteries (ICA) and vertebral arteries (VA)

Biffl grade	ICA	VA	Total
I	20	5	25
II	15	8	23
III	6	2	8
IV	8	7	15
V	0	1	1
			72

Interobserver agreement was overall substantial (Kappa = 0.674). In 41 of all cases, reviewers disagreed initially (11%). Differences concerned predominantly Grade I injuries (*n* = 25; 6.7%), thereof 13 false negative (3.5%) and 5 false positive lesions (1.3%). In grade II injuries, there was initial disagreement in 17 cases (4.5%), thereof 9 false negative (2.4%) and 4 false positive findings (1.1%). There was no interobserver disagreement for Biffl grades III through V.

Based on patients’ documented clinical findings and concomitant injuries on WBCT, 71 patients would have met the standard Denver criteria, of which 24 (34%) suffered a BCVI. BCVI was found in 32 (11%) of the 303 patients who would not have met these criteria.

**Table 6:** Number of BCVI by vertebral body level in internal carotid arteries (ICA) and vertebral arteries (VA)

BCVI level	ICA	VA	Total
C0	8	1	9
C1	14	3	17
C2	16	4	20
C3	6	3	9
C4	5	5	10
C5	0	1	1
C6	0	1	1
C7	0	3	3
Th1	0	2	2
			72

Concomitant injuries affected most commonly the chest (66%), followed by head injuries (46%) and abdominal injuries (30%). Cervical spine and pelvic area were affected with 26% and 25%, respectively (Table 4).

**Fig. 6:**
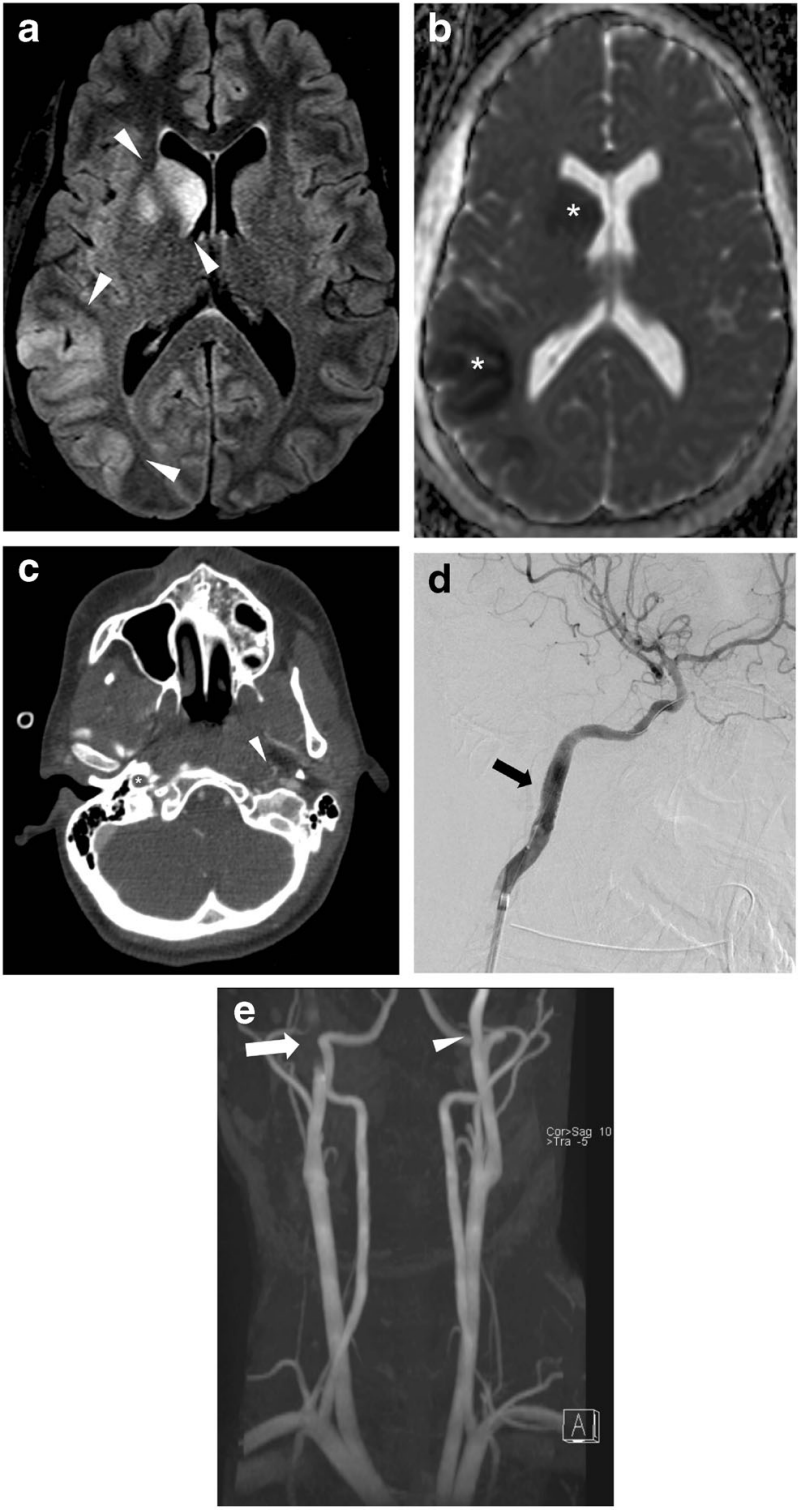
Twenty one-year-old female with polytrauma after MVA; BCVI of both ICA. **a** Ischemic lesion and focal swelling in the area of the right caudate nucleus and lentiform nucleus, with an additional ischemic lesion in the right parietal cortex (1.5 T MRI; FLAIR). **b B**oth lesions show decreased diffusion (arrowheads; 1.5 T MRI; DWI). **c** Grade IV BCVI of the right ICA (asterisk), and grade II lesion of the left ICA on the level of the skull base (arrowhead) on axial CTA images. Both VA remain open. **d** Wire stent of the right ICA placed under fluoroscopy guidance on DSA summation image (arrow). **e** 1.5 T MRA of the craniocervical arteries after stent placement demonstrating near normal patency. Signal loss caused by the metallic wire stent (arrow) in the right ICA. Residual grade II BCVI lesion of the left ICA (arrowhead)

Two hundred fifteen of the 374 patients subjected to CTA (57.4%) underwent brain imaging by either CT (*n* = 105; 48.8%), MRI (*n* = 38; 17.7%), or both CT and MRI (*n* = 72; 33.5%) up to 53 months after the initial trauma. The mean time from initial admission to repeat cranial imaging by MRI or CT was 147 days for people with BCVI, and 138 days for people without BCVI, with a median of 1 day for both groups. The majority of cases was imaged the following day (with BCVI *n* = 18; 47%, and without BCVI *n* = 71; 40%). Indications for imaging ranged from primary follow-up studies for the same initial trauma to newly acquired trauma, and also included imaging for unrelated conditions (Fig. 6).

There were cerebral ischemic lesions in 4% (*n* = 15) of all patients, with an incidence of *n* = 9 in BCVI patients (16.1%) and *n* = 6 in patients without BCVI (1.9%). Fisher’s exact test shows the difference in incidence between these groups to be statistically significant (*p* < .0001), with an odds ratio of 9.77 (95% CI 3.3–28.7). Of the 9 BCVI patients who developed an ischemic lesion, 3 (3/9; 33.3%) would have met the Denver criteria, while 6 (6/9; 66.7%) would not. Conversely, of the 6 patients with ischemic lesions and no BCVI finding, 1 (1/6; 16.7%) would have met the Denver criteria, and 5 (5/6; 83.3%) would not.

## Discussion

Applying our augmented imaging criteria resulted in a markedly higher detection rate for BCVI compared to previous reports [3, 12, 13, 15, 17] while being high in the previously reported range with 3.6% when calculated based on the overall number of trauma patients, which indicates a favorable rate of selection for screening. This is in accordance with publications predicting a higher incidence of BCVI with more liberal screening [22, 27, 28].

Since the exact trauma mechanism may be difficult or even impossible to determine with certainty during the initial phase, this particular criterion might not be applicable for every patient. Correct recognition of clinical criteria on the other hand depends on the ER physician’s experience, as well as on the patient’s ability to cooperate. BCVI will often remain initially clinically silent or might be masked by concomitant injury or intoxication.

When retrospectively applying Denver screening criteria, 11% of BCVI patients in our cohort would not have been imaged, thereby resulting in these BCVI being missed with potentially disastrous consequences.

The distribution of BCVI follows the mechanical properties of the cervical spine and occipito-cervical junction. The highest incidence of BCVI in ICA was on the level of C2, where high mobility facilitates stretching and rotation, which are the major factors in the etiology of BCVI [1, 2, 29]. In the VA, BCVI is more evenly distributed, likely due to the support provided by the transverse foramina. The most common injury grades were grades I and II in the ICA and grades II and IV in the VA. The low number of grade III injuries in the VA might be due to the relatively small caliber of the transverse foramina, which might predispose for vessel occlusion rather than pseudoaneurysm formation. The singular grade V injury in our cohort does not reflect an actual low incidence, but rather the very high mortality rate associated with these injuries.

The predominant mechanisms of injury MVA, fall from a height, bicycle accident, and pedestrian in traffic represent the most common causes for ER admission due to blunt trauma, highlighting the broad spectrum of possible mechanisms leading to BCVI. Any high-energy deceleration event can potentially cause extension, flexion or rotation of the neck because of the inertia of the head, and warrants therefore inclusion into BCVI screening criteria. Blunt trauma to the chest is the second most common cause of death in polytrauma patients after CNS trauma, and may indicate that major deformation of the thoracic area has occurred during the time of impact [30], with the possibility of distortion of the neck.

The downside of including CTA in WBCT is slightly decreased image quality due to patients’ arms being raised during the scan in order to reduce imaging artifacts as well as radiation dose to the chest and abdomen, leading to increased image noise and imaging artifacts in the neck.

Interobserver agreement for BCVI was substantial with kappa = 0.674. Disagreements affected mainly grade I and II injuries, which are minimal lesions in the vessel walls and are especially hard to detect in the presence of image noise, artifacts, medical equipment, and concomitant injury. CTA as part of the initial WBCT has been shown to have a sensitivity up to 90% in more recent publications [25], with older articles claiming sensitivities of greater than 97% with a specificity of over 86% [31, 32]. Extending the contrast-enhanced scan to the skull base and neck takes only a minimal amount of additional time, especially if otherwise a non-enhanced CT of the cervical spine would have been performed. The high interobserver agreement for BCVI implies that inclusion of the cervical arteries into routine WBCT is an effective means of identifying BCVI in trauma patients.

The marked increase in ischemic CNS lesions found on cranial imaging in BCVI patients after the initial trauma compared to those without BCVI findings shows the delayed effects acute vascular injuries may have, and that these may occur even years after the initial trauma. Even though a direct correlation between the documented vascular injury and subsequent CNS ischemia cannot be established based on our data, there is a significant relative risk associated with previous BCVI. This emphasizes the importance of detecting these injuries to allow for adequate treatment.

Concomitant injury was most frequently found in the area of the chest at 66%, which emphasizes the importance of including injury to this area into the criteria for screening. Cervical spine injuries, somehow counterintuitively, were encountered by comparison considerably less frequently coinciding with BCVI at 26%. Also, head injury with 46% appears to be a more reliable indicator of BCVI than injury to the cervical spine.

When applying Denver criteria retrospectively to our data, the number to scan to find a BCVI in asymptomatic patients would be ten. Most likely, these patients would not have been imaged according to standard criteria, and their injuries would therefore almost certainly have been missed initially.

Limitations of this study include that due to it being retrospective, documentation was not completely uniform, and information like injury severity score (ISS) and Glasgow coma scale (GCS) could not be retrieved for all patients. Also, there was no DSA confirmation of either positive or negative findings, so specificity and sensitivity could not be calculated. Cranial imaging was not standardized for follow-up of the primary vascular lesions, and the etiology of ischemic lesions is impossible to trace conclusively to the primary trauma.

In conclusion, based on the high detection rate of BCVI using our modified Denver criteria, we recommend including CTA into WBCT for trauma in any high-energy deceleration trauma to the chest, abdomen, or pelvis in addition to the basic Denver criteria.
